# Communicative health literacy and associated variables in nine European countries: results from the HLS_19_ survey

**DOI:** 10.1038/s41598-024-79327-w

**Published:** 2024-12-04

**Authors:** Salvatore Metanmo, Hanne Søberg Finbråten, Henrik Bøggild, Peter Nowak, Robert Griebler, Øystein Guttersrud, Éva Bíró, Brigid Unim, Rana Charafeddine, Lennert Griese, Zdenek Kucera, Christopher Le, Doris Schaeffer, Mitja Vrdelja, Julien Mancini, The HLS19 Consortium, Jürgen Pelikan, Christa Straßmayr, Robert Griebler, Christina Dietscher, Stephan van den Broucke, Rana Charafeddine, Antoniya Yanakieva, Nigyar Dzhafer, Zdeněk Kucera, Alena Steflova, Henrik Bøggild, Andreas Jull Sørensen, Julien Mancini, Cécile Allaire, Doris Schaeffer, Alexander Schmidt-Gernig, Éva Bíró, Lucy Bruton, Sarah Gibney, Diane Levin-Zamir, Luigi Palmieri, Daniela Galeone, Kjell Sverre Pettersen, Christopher Le, Andreia Jorge Silva da Costa, Miguel Telo de Arriaga, Maria Lopatina, Oxana Drapkina, Zuzana Klocháňová, Mitja Vrdelja, Tamara Štemberger Kolnik, Saskia Maria De Gani, Karin Gasser

**Affiliations:** 1 INSERM, IRD, ISSPAM, SESSTIM, Cancer, Biomedicine & Society Group, Ligue 2019 Accredited Team, Aix Marseille Univ, 13009 Marseille, France; 2 Department of Health and Nursing Sciences, Faculty of Social and Health Sciences, Inland Norway University of Applied Sciences, P.O. Box 400, 2418 Elverum, Norway; 3 Public Health and Epidemiology, Department of Health Science and Technology, Aalborg University, 9220 Aalborg, Denmark; 4 Competence Centre for Health Promotion and Health System, Austrian National Public Health Institute, 1010 Vienna, Austria; 5 Norwegian Centre for Science Education, Faculty of Mathematics and Natural Sciences, University of Oslo, Blindern, PO Box 1106, 0317 Oslo, Norway; 6 Department of Public Health and Epidemiology, Faculty of Medicine, University of Debrecen, 4028 Debrecen, Hungary; 7 Department of Cardiovascular, Endocrine-Metabolic Diseases and Aging, Istituto Superiore Di Sanità, 00162 Rome, Italy; 8 Department of Epidemiology and Public Health, Sciensano, Brussels, Belgium; 9 School of Public Health, Bielefeld University, 33615 Bielefeld, Germany; 10 Czech Health Literacy Institute, Sokolská 490/31, 120 00 Prague, Czech Republic; 11 Communication Unit, National Institute of Public Health, Trubarjeva 2, 1000 Ljubljana, Slovenia; 12 APHM, Public Health Department (BIOSTIC), 13005 Marseille, France; 13 UMR1252 SESSTIM (Aix-Marseille Univ), Institut Paoli-Calmettes, 232 Bd Ste Marguerite, BP 156, 13273 Marseille Cedex 9, France; 14 Competence Centre Health Promotion and Healthcare, Austrian National Public Health Institute, 1010 Vienna, Austria; 15 Austrian Ministry of Labour, Social Affairs, Health and Consumer Protection, Vienna, Austria; 16 Psychological Sciences Research Institute, Université Catholique de Louvain, 1348 Louvain-la-Neuve, Belgium; 17 Department of Public Health and Epidemiology, Sciensano, 1050 Brussels, Belgium; 18 Faculty of Public Health, Medical University, Sofia, Bulgaria; 19 Bulgarian National Assembly, Sofia, Bulgaria; 20 Ministry of Health of the Czech Republic, Prague, Czech Republic; 21 Ministry of Health, Copenhagen, Denmark; 22 Department of Public Health, SESSTIM, Aix Marseille Univ, Inserm, IRD, APHM, Marseille, France; 23 Santé Publique France, Saint-Maurice, France; 24 Federal Ministry of Health, Berlin, Germany; 25 Department of Health, Dublin 2, DO2 XW14 Ireland; 26 Clalit Health Services, Ministry of Health University of Haifa, Haifa, Israel; 27 Istituto Superiore di Sanità-ISS, Rome, Italy; 28 Ministry of Health, Rome, Italy; 29 Oslo Metropolitan University, Oslo, Norway; 30 Norwegian Directorate of Health, Oslo, Norway; 31 Directorate-General of Health, Lisbon, Portugal; 32 National Medical Research Center for Therapy and Preventive Medicine under the Ministry of Health of the Russian Federation, Moscow, Russian Federation; 33 National Medical Research Center for Preventive Medicine under the Ministry of Health of the Russian Federation, Moscow, Russian Federation; 34 Public Health Authority of the Slovak Republic, Bratislava, Slovakia; 35 Ministry of Health Slowenia, Directorate for Public Health, Ljubljana, Slovenia; 36 Careum Foundation, Careum Center for Health Literacy, 8032 Zurich, Switzerland; 37 Federal Office of Public Health, Bern, Switzerland

**Keywords:** Communicative health literacy, HLS_19_, Socio-economic status, Health disparities, Physician–patient communication, Epidemiology, Public health

## Abstract

Our study aimed to report on variables associated with communicative health literacy (COM-HL) in European adults. The HLS_19_ survey was conducted in 2019–2021 including nine countries which measured COM-HL by using a validated questionnaire (HLS_19_-COM-P-Q6 with a score ranging from 0 to 100). Linear regression models were used to study variables associated with COM-HL globally (multilevel model with random intercepts and slopes and at country level) and in each country. Additional models studied each of the HLS_19_-COM-P-Q6 items separately. The mean COM-HL score ranged between 62.5 and 76.6 across countries. Among the 18,137 pooled participants, COM-HL was positively associated with age, a higher self-perceived social status, previous training in healthcare, an increasing number of general practitioner visits; and negatively associated with female sex, reported financial difficulties, having a chronic condition and an increasing number of specialist visits. These effects were heterogeneous from one country to another, and from one item to another when analysing the different COM-HL items separately. However, there was a consistent statistically significant association between COM-HL (score and each item) and financial difficulties as well as self-perceived social status in all countries. Interventions to improve communication between patients and physicians should be a high priority to limit communication disparities.

## Introduction

Health literacy (HL) entails the skill and motivation that enable individuals to obtain, understand, evaluate and use information to make decisions and take actions that affect their health and wellbeing^1,2^. The modernization and digitalization of healthcare, with increasing patient empowerment ^3^, places greater demands on patients’ HL and potentially leads to health disparities ^4^. One of the crucial elements for relating to the healthcare system is the ability to communicate with healthcare professionals, especially physicians. To estimate the level of HL in populations, the World Health Organization (WHO) recommends regular, standardized measurement of HL in the general population^5^. This was done—as a first step—in the Health Literacy Survey 2019–2021 (HLS_19_) conducted by the WHO Action Network on Measuring Population and Organizational Health Literacy (M-POHL) in the WHO European region^6^. “Communicative Health Literacy (COM-HL)”, a specific aspect of HL, was also assessed in HLS_19_ survey.

COM-HL “refers to patients’ (the term is used as a synonym for healthcare users, clients, citizens, individuals, and people) communicative and social skills that enable them to actively engage in face-to-face encounters with healthcare professionals, to give and seek information, derive meaning from it, and apply this information in decision making and in co-producing their health care”^7^ (p. 235). COM-HL is therefore a key aspect of HL, and improving the quality of communication between patients and their healthcare professionals is a critical factor in promoting health^8,9^. Clarifying their message by limiting the amount of information, using plain language^10^, presenting recommendations in discrete action-oriented steps and evaluating comprehension are some of the techniques suggested for improving communication by clinicians^11^.

It has already been established that general HL is a determinant of health, regardless of the indicator considered, for instance: mortality^12^, compliance with treatment ^13^, self-efficacy and positive health behaviours, and glucose monitoring in chronic diseases such as diabetes^14,15^. However, there are few specific publications on COM-HL^7^, although better health communication can have a positive impact on health events, but also on prevention and health promotion^15^. Identifying the factors associated with COM-HL would provide healthcare players (i.e., health administration and organizations, medical staff) and policy-makers levers to effectively improve the level of COM-HL in populations or to target interventions to those more susceptible to difficulties. In particular, the existence of a social gradient is well known for general HL^2^. However, as HL results from personal abilities and system complexity, COM-HL might be less affected by social characteristics if physicians are more able to adapt their communication. The aim of this study was to report on the level of COM-HL and on the sociodemographic and Health related variables associated with COM-HL in the general population of nine countries in Europe.

## Methods

### Type and context of study

We collected data as part of the European cross-sectional survey HLS_19_, which took place between December 2019 and June 2021. Of the 17 countries that participated in this survey, the nine that used the short version of the optional COM-HL scale (HLS_19_-COM-P-Q6) were: Austria (AT), Belgium (BE), Bulgaria (BG), Czech Republic (CZ), Germany (DE), Denmark (DK), France (FR), Hungary (HU) and Slovenia (SI)^7^. HLS_19_ was a general population survey based on multi-stage random sampling or quota sampling procedures in most countries^6^. The type of sampling, method of data collection, and number of participants are described in Table 1.

**Table 1:** Sociodemographic, health characteristics and communicative health literacy of the study sample by country.

Characteristic^*1*^	AT, n = 2819	BE, n = 1000	BG, n = 682	CZ, n = 1572	DE, n = 2103	DK, n = 3575	FR, n = 2003	HU, n = 1123	SI, n = 3261	Overall, n = 18,137	p-value^*2*^
Mode of data collection	CATI	CAWI	CAPI, CAWI	CATI, CAWI	PAPI	CAWI	CAWI	CATI	CAPI, CAWI	NA	NA
Sampling procedure	Multi-stage random sampling	Quota sampling	Proportional stratified sampling (CAPI) and random quota sampling (CAWI)	Random digital procedure (CATI) and random quota sampling (CAWI)	Multi-stage random and quota sampling combined	Multi-stage random sampling	Quota sampling	Multi-stage random sampling	Multi-stage random sampling	NA	NA
Age (years)*, mean (SD)*	48.7 *(17.8)*	47.8 *(16.1)*	47.6 *(17.4)*	48.3 *(17.0)*	50.7 *(18.4)*	51.6 *(18.2)*	46.1 *(15.7)*	47.9 *(17.5)*	50.1 *(18.1)*	49.3 *(17.7)*	** < 0.001**
* Unknown*	*4*	*0*	*0*	*0*	*18*	*0*	*0*	*0*	*0*	*22*	
Sex (female)	51%	50%	54%	51%	51%	53%	51%	53%	50%	51%	0.650
* Unknown*	*0*	*0*	*0*	*0*	*6*	*0*	*0*	*0*	*0*	*6*	
Rural living	38%	30%	27%	26%	20%	33%	30%	47%	47%	35%	** < 0.001**
* Unknown*	*0*	*0*	*0*	*0*	*0*	*0*	*1*	*0*	*0*	*1*	
Born abroad	7.2%	3.3%	1.7%	3.4%	5.7%	5.7%	4.3%	3.1%	13%	6.5%	** < 0.001**
* Unknown*	*2*	*3*	*0*	*0*	*6*	*0*	*0*	*0*	*2*	*13*	
Level of education											** < 0.001**
Lower secondary education	18%	3.0%	7.9%	44%	11%	9.8%	3.6%	50%	33%	20%	
Upper secondary education	49%	12%	23%	32%	48%	8.7%	14%	26%	38%	29%	
≤ Bachelor’s degree	16%	41%	11%	4.0%	12%	38%	44%	8.0%	8.3%	21%	
≥ Bachelor’s degree	17%	44%	58%	20%	28%	44%	39%	16%	21%	30%	
* Unknown*	*0*	*12*	*6*	*1*	*41*	*5*	*0*	*0*	*0*	*64*	
Trained in a health profession	13%	18%	34%	6.8%	9.8%	17%	20%	22%	8.2%	14%	** < 0.001**
* Unknown*	*17*	*0*	*7*	*0*	*9*	*3*	*0*	*1*	*2*	*37*	
Financial difficulties (ability to pay bills)											** < 0.001**
Very difficult	2%	9%	4%	4%	3%	1%	3%	5%	7%	3%	
Difficult	13%	29%	32%	25%	21%	7%	23%	28%	35%	21%	
Easy	51%	42%	51%	55%	56%	47%	55%	57%	52%	52%	
Very easy	35%	21%	13%	17%	20%	45%	20%	9%	6%	24%	
* Unknown*	*33*	*0*	*17*	*2*	*86*	*7*	*0*	*19*	*26*	*189*	
Self-perceived social status*, mean (SD)*	6.2 *(1.5)*	6.5 *(1.5)*	6.1 *(1.7)*	5.7 *(1.6)*	5.9 *(1.6)*	6.3 *(1.7)*	5.6 *(1.5)*	5.1 *(1.5)*	5.4 *(1.6)*	5.9 *(1.6)*	** < 0.001**
* Unknown*	*151*	*0*	*82*	*10*	*55*	*11*	*0*	*11*	*62*	*382*	
Have one chronic condition or more	37%	48%	52%	60%	51%	51%	45%	43%	40%	46%	** < 0.001**
* Unknown*	*8*	*0*	*17*	*0*	*36*	*4*	*0*	*4*	*7*	*75*	
Number of visits to the GP											** < 0.001**
0	22%	9.9%	28%	28%	14%	18%	14%	24%	30%	21%	
1	22%	20%	21%	23%	17%	24%	21%	16%	24%	21%	
2	21%	19%	15%	19%	20%	21%	19%	12%	18%	19%	
≥ 3	36%	51%	37%	30%	49%	37%	46%	48%	28%	38%	
* Unknown*	*65*	*7*	*0*	*19*	*72*	*10*	*0*	*1*	*11*	*185*	
Number of visits to a specialist											** < 0.001**
0	26%	33%	53%	28%	31%	64%	36%	41%	51%	42%	
1	23%	24%	22%	20%	20%	17%	24%	19%	25%	21%	
2	20%	17%	10%	19%	18%	9.6%	17%	15%	11%	15%	
≥ 3	30%	25%	15%	33%	31%	9.8%	22%	25%	14%	22%	
* Unknown*	*30*	*17*	*0*	*20*	*40*	*10*	*0*	*1*	*18*	*135*	
COM-HL score*, mean (SD)*	76.6 *(16.8)*	72.6 *(19.9)*	64.8 *(17.0)*	68.3 *(18.9)*	62.5 *(17.9)*	69.0 *(19.2)*	68.3 *(18.9)*	69.1 *(16.1)*	73.8 *(17.1)*	70.2 *(18.5)*	** < 0.001**
Missing data for COM-HL	5.0%	0%	21%	1.7%	1.9%	0.7%	0%	6.0%	2.6%	3.1%	** < 0.001**

^*1*^Data are n or % unless otherwise specified, ^*2*^Wilcoxon rank-sum test for complex survey samples; chi-squared test with Rao & Scott’s second-order correction; AT: Austria; BE: Belgium; BG: Bulgaria; CAPI: computer-assisted personal interviews; CATI: computer-assisted telephone interviews; CAWI: computer-assisted web interviews; COM-HL: communicative health literacy; CZ: Czech Republic; DK: Denmark; FR: France; DE: Germany; GP: general practitioner; HU: Hungary; PAPI: paper-assisted personal interviews; NA: Not applicable; SI: Slovenia.

Significant p-values are in bold.

### A measure of communicative health literacy in patient–physician communication: the HLS_19_-COM-P-Q6

The HLS_19_-COM-P-Q6 was developed for the purpose of the HLS_19_ study. It has been validated using confirmatory factor analysis and Rasch analysis, has good psychometric properties and can be used to measure COM-HL, regardless of the data collection mode^16^. The six questions focusing on patient-physician communication are: On a scale from very easy to very difficult, how easy would you say it is …:

- …to explain your health concerns to your doctor? (COM1)
- …to get enough time in the consultation with your doctor? (COM2)
- …to express your personal views and preferences to your doctor? (COM3)
- …to ask your doctor questions in the consultation? (COM4)
- …to be involved in decisions about your health in dialogue with your doctor? (COM5)
- …to recall the information you get from your doctor? (COM6)

Note that the item numbering is not the same as in previous publications^7,16^, which were based on the original long version (11 items) of the scale. For each of the six items, the level of difficulty was rated with one of the following four responses: “very easy” (4), “easy” (3), “difficult” (2) and “very difficult” (1). Based on the responses provided, polytomous total scores were calculated for each participant, initially ranging from 6 to 24. To make it easier to understand the results, we then linearly transformed this raw score to a value between 0 and 100 ([raw score-6]*100/18), with a higher value indicating higher COM-HL^17^.

### Other survey questions

Other variables collected included socio-demographic characteristics such as age, sex, migration background (a 4-point ordinal variable was used with the following response options: “none”, “one parent was born abroad”, “both parents were born abroad”, “born abroad”) and type of area of residence (urban or rural). Socio-economic status was assessed by level of education, financial difficulties (ability to pay bills; answers ranging from “1 = very difficult” to “4 = very easy”), and perceived social status, ranging from 1 (lowest self-assessed level in society) to 10 (highest level in society). Health related variables, such as the presence of a chronic condition, the number of visits to a general practitioner (GP) or specialist, and previous training in a health profession were also collected. More details on the collection of these variables are available in the HLS_19_ International Report^6^.

### Data collection

Each participating country used one or more of the following four methods for data collection: CAPI = computer-assisted personal interviews; CATI = computer-assisted telephone interviews; CAWI = computer-assisted web interviews; PAPI = paper-assisted personal interviews. BG, CZ, and SI combined different methods of data collection. For this study, we combined CAPI and PAPI, so that three modes were considered: CAPI/PAPI (face-to-face interviews), CATI (telephone interviews), and CAWI (self-administered online surveys). Twelve Slovenian participants were excluded from all the analyses below because the mode of data collection (self-administered with paper and pencil) did not correspond to any of the four previous modes.

### Statistical analysis

All analyses were performed using R version 4.3.1, and, due to the stratified sampling of participants, were weighted by age, sex, and several other characteristics (area of residence and its population density, or education level) across countries.

Descriptive analysis was carried out to describe the sample and the COM-HL level of the population in all and then in each participating country. The participants (n = 585; 3.1%) with at least one missing item for one of the six items of the HLS_19_-COM-P-Q6 were excluded from the analyses. Their characteristics were also described. Wilcoxon rank-sum test for complex survey samples and chi-squared test were used to compare variables according to their type.

#### Variables associated with COM-HL scale score

To study the factors associated with the COM-HL score, we used multilevel models. We first built a M0 (initial) model with an intercept and a random effect for country to estimate the intra-class coefficient (ICC) for the country effect. A multivariable model (M1) was then estimated introducing all candidate variables and random intercepts at country level. Then, for each variable, a random slope was added when the Akaïke criterion (AIC) was significantly improved. Thus, the final conservative M2 model included random slopes for all variables except training in health profession and number of specialist visits. Linear regression models were then used to examine the same multivariable model in each country. For each model, the assumptions of the linear model were checked. In particular, the homoscedasticity, independence and normality of the residuals were verified.

#### Variables associated with each COM-HL item

An analysis of associated variables was also conducted for each COM-HL item. For this, the odds of answering "difficult/very difficult" to the different items were estimated using multilevel binary logistic regression (with random intercepts at country level). Such as for the score, we added random slopes when AIC improved.

All models were adjusted for the mode of data collection, and for all multilevel models, the ICC for the country effect was reported.

#### Modelling quantitative variables

We categorized age using its deciles. Since the effect of age was not linear for all items, we decided to use age in categories (provided by quartiles) in all the models in this study. Self-perceived social status is included quantitatively in the models. Finally, the number of visits to a GP or specialist was treated from the outset as a four-category variable.

### Ethical considerations

Ethical requirements for health research have been met in each country in accordance with the Declaration of Helsinki. We confirm that all experiments were performed in accordance with relevant guidelines and regulations. In each country, informed consent was obtained from each study participant. In countries where national regulations so require, ethical approval has been obtained from an ethics committee. In France, this study was approved by the Ethics Evaluation Committee of the French National Health and Medical Research Institute (CEEI, IRB 00003888). More detailed information on ethical considerations, data protection and informed consent by country is available in the HLS19 International Report^6^.

## Results

### Sample characteristics

The mean age of the study sample (n = 18,137) was 49.3 years (standard deviation (SD): 17.7), and 51% were female. The majority of the sample lived in urban areas (65%) and about a third had a bachelor’s degree or higher (30%). A minority (6.5%) were foreign-born, and only a few had been trained in a health profession (14%). The mean self-perceived social status was 5.9/10 (SD: 1.7) (Median 6 (IQR; 5, 7)). Regarding financial difficulties, three quarters found it easy or very easy to pay their bills, while 3.4% found it very difficult. A significant proportion (46%) of the sample had at least one chronic condition. In the last 12 months, many respondents reported regular (≥ 3) visits to a GP (38%) and at least one visit to a specialist (58%). Details of these characteristics and their distribution by country are shown in Table 1.

The frequency of missing data was similar across the six HLS_19_-COM-P-Q6 items, ranging from 0.5% (COM1) to 1.3% (COM6). We compared the characteristics of the included versus the excluded population with at least one missing HLS_19_-COM-P-Q6 item (Tables S1, S2). Excluded participants were older, less educated, reported a lower self-perceived social status, and were less likely to visit their GP. Data collection by CATI also significantly increased the odds (adjusted OR = 24.6 (95% CI: 3.16, 19)) of missing the HLS_19_-COM-P-Q6 item.

### Communicative health literacy in the adult European population

The mean COM-HL score was heterogeneous across countries, ranging from 62.5 to 76.6 (Table 1).

**Fig. 1:**
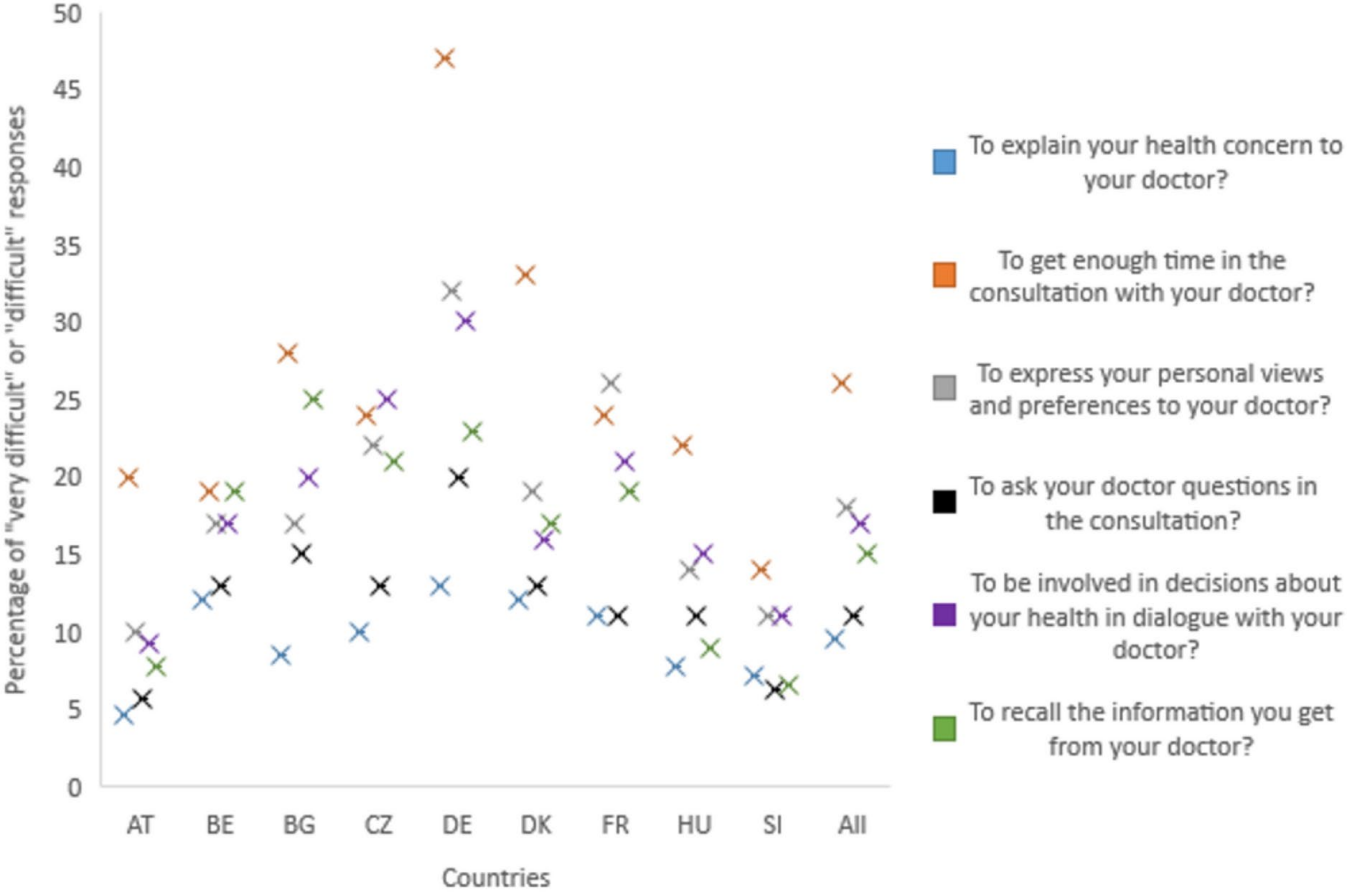
shows the percentage of “difficult” responses for each item by country. Difficult" and “very difficult” have been grouped together. Items are represented by crosses with the corresponding colour in the legend. The mean value for each item is presented after all countries. Item COM1 (expressing health concerns…) was the least difficult in all countries; item COM2 (finding time…) was the most difficult in all countries except the Czech Republic (CZ) and France (FR), where the most difficult items were COM5 (being involved in decisions…) and COM3 (expressing preferences…) respectively.

Figure 1 shows the percentage of “very difficult” and “difficult” responses by country. Item COM1 (to explain your health concerns to your doctor?) appears to be the least difficult in all countries, while item COM2 (to get enough time in the consultation with your doctor?) is the most difficult. Exceptions are the Czech Republic and France, where the most difficult items were COM5 (to be involved in decisions about your health in dialogue with your doctor?) and COM3 (to express your personal views and preferences to your doctor?), respectively. Difficulties varied from one item to item and from one country to country, with the percentage of difficulties reported ranging from 4.6 to 47%.

### Factors associated with communicative health literacy

#### COM-HL score

Table 2 shows the factors associated with the COM-HL score. In bivariate analysis, all considered variables were statistically significantly associated with COM-HL. After multivariate adjustment, higher education and urban residence were no longer statistically significant. Age, self-perceived social status, being trained in a health profession and number of visits to a GP were positively associated with COM-HL. Conversely, being a woman, having financial difficulties, a chronic condition and more visits to specialists were negatively associated with COM-HL.

**Table 2:** Variables associated with communicative health literacy (COM-HL) score (multilevel models).

Variables	Initial model*		Final multivariate model*	
	β coef.	*p-value*	β coef.	*p-value*
Age (years)**				
≤ 34	Ref		Ref	
]34; 50]	0.00	0.993	0.21	0.8
]50; 64]	1.14	0.002	2.30	**0.047**
> 64	0.83	0.034	1.90	0.2
Sex				
Male	Ref		Ref	
Female	− 1.20	< 0.001	− 0.76	**0.005**
Area of residence**				
Urban	Ref		Ref	
Rural	− 0.64	0.024	− 0.21	0.7
Level of education**				
≤ Lower secondary education	Ref		Ref	
Upper secondary education	1.88	< 0.001	− 0.63	0.4
≤ Bachelor’s degree	2.07	< 0.001	− 1.2	0.3
≥ Bachelor’s degree	3.68	< 0.001	− 2.0	0.12
Financial difficulties (ability to pay bills)**				
Very easy	Ref		Ref	
Easy	− 8.57	< 0.001	− 6.9	** < 0.001**
Difficult	− 12.39	< 0.001	− 9.7	** < 0.001**
Very difficult	− 12.71	< 0.001	− 9.5	**0.003**
Self-perceived social status**	2.01	< 0.001	1.3	** < 0.001**
Trained in a health profession				
Yes	Ref		Ref	
No	− 4.11	< 0.001	− 3.8	** < 0.001**
Chronic condition**				
No	Ref		Ref	
Yes	− 2.16	< 0.001	− 1.4	** < 0.029**
Number of visits to the GP**				
0	Ref		Ref	
1	1.28	0.002	1.5	**0.014**
2	0.66	0.123	1.3	0.053
≥ 3	− 0.12	0.755	1.4	0.11
Number of visits to a specialist				
0	Ref		Ref	
1	− 0.17	0.641	− 0.51	0.077
2	− 1.12	0.007	− 1.26	**0.002**
≥ 3	− 1.83	< 0.001	− 1.25	**0.003**
Country effect (ICC)	5%***		11%	

*All models account for the clustering (country) effect and are adjusted for mode of data collection. ICC: intra-class correlation coefficient; GP: general practitioner. **Random slope at country level was added in the multilevel model; ***The initial model with only intercept and a random effect for country was statistically significant (p < 0.001).

Significant p-values are in bold.

Table 3 summarizes the type of association (positive, negative, not significant) between each independent variable and the COM-HL score in each country. Financial difficulties and self-perceived social status were the only variables that were statistically significantly associated in the same direction with the COM-HL score in all countries. The details of the variables associated with the COM-HL score in each country are presented in the appendices (Figs. S1 to S9).

**Table 3:**
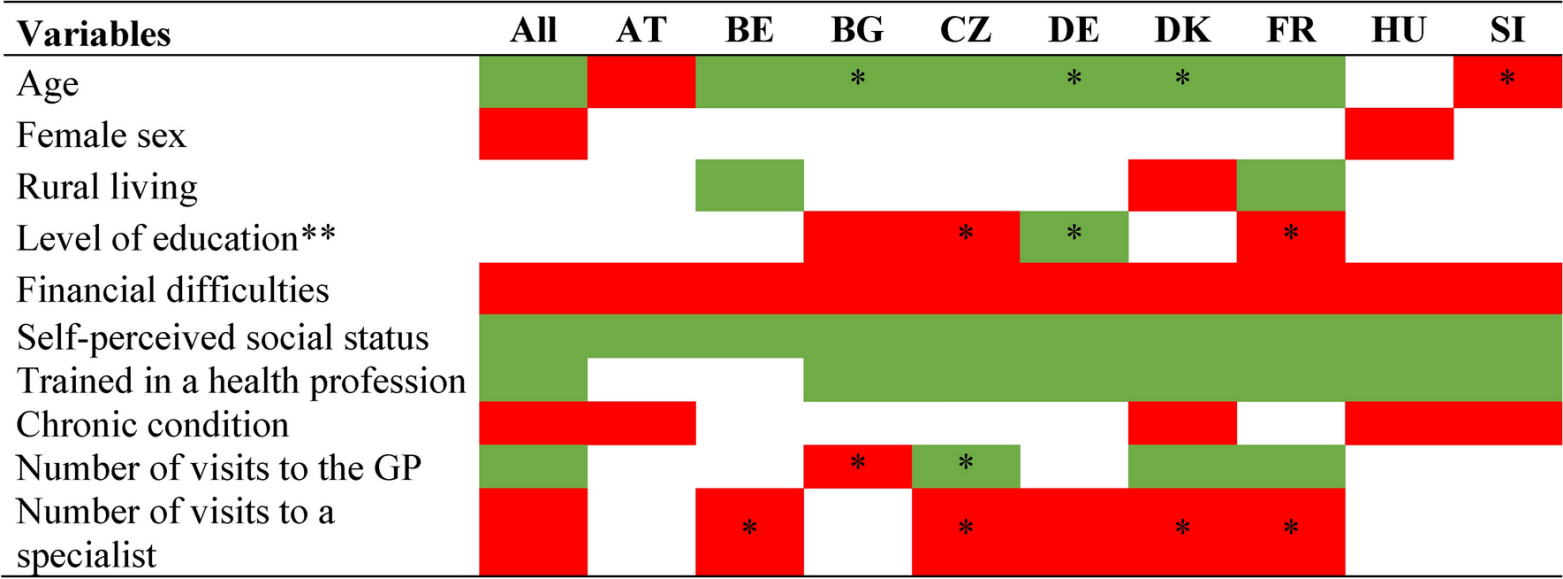
Associations between variables and the communicative health literacy (COM-HL) score in HLS_19_ survey.

*The p-value is statistically significant for a single class of the categorical variable. ** Reverse association might be observed after multiple adjustments. AT: Austria; BE: Belgium; BG: Bulgaria; CZ: Czech Republic; DK: Denmark; FR: France; DE: Germany; GP: general practitioner; HU: Hungary; SI: Slovenia; HLS_19_: Health Literacy Survey 2019–2021; To investigate the variables associated with the COM-HL score, multilevel model was used for the pooled data (All) and multiple linear regression models was used in each country.

#### Factors associated with each COM-HL item

Table 4 shows the direction of associations between each independent variable and the responses for a given item (cf. Tables S3 to S8 in the Appendix for details). The size of the effect could vary depending on the item, but there were no significant reverse effects. Older age, lack of financial difficulties, higher self-perceived social status, and lack of chronic condition were significantly associated with fewer difficulties for all items, while the other variables were only significantly associated with certain items. Female sex was associated with more difficulties, except for difficulties recalling the information received (COM6); living in a rural area was associated only with the ease of getting more time for consultation (COM2); the negative effect of higher education was significant only for getting enough time for consultation (COM2) and being involved in decisions (COM5); training in a health profession did not facilitate getting enough time for consultation (COM2), expressing personal views and preferences (COM3), and asking questions (COM4). The pattern of association was almost the opposite for the number of consultations with GP (facilitating getting time, asking questions, being involved in decisions, and recalling information) or specialists (limiting explaining concerns, getting time, and expressing personal views).

**Table 4:**
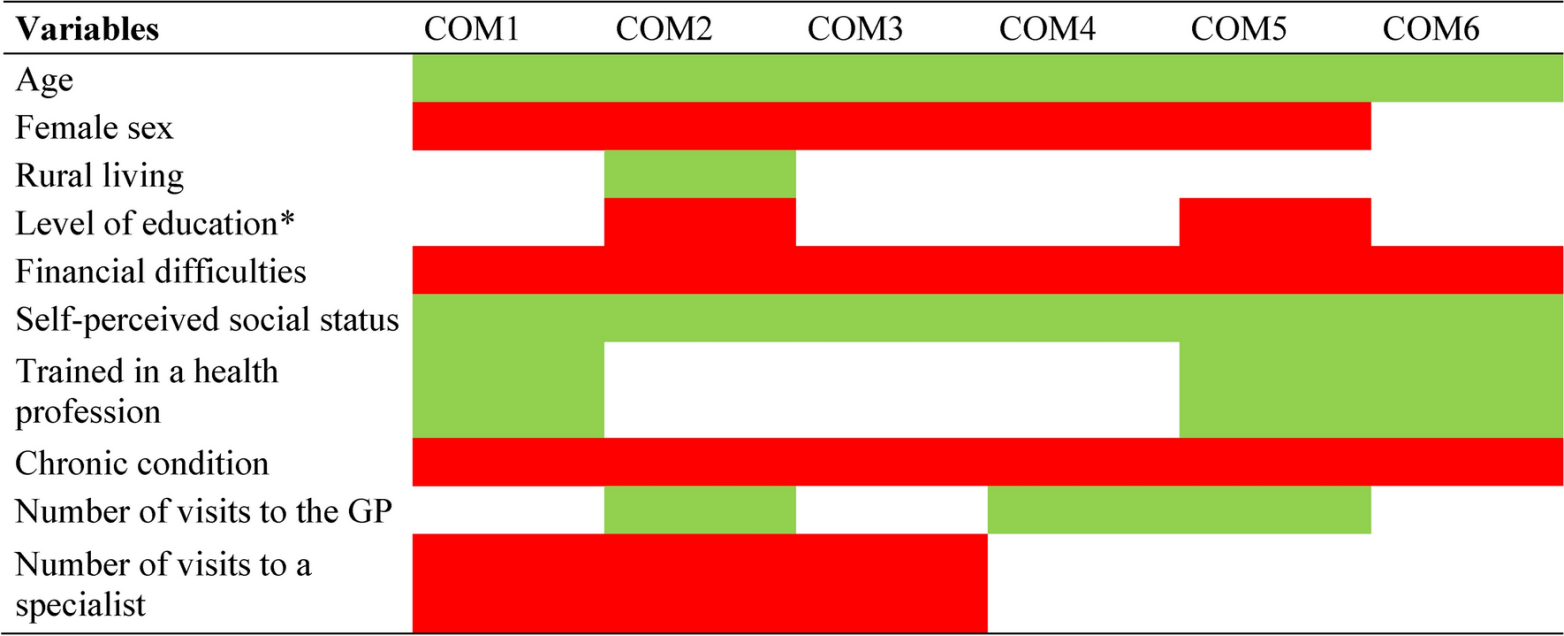
Summary of the direction of associations between each item and the variables (multilevel multivariable binary logistic regression models).

*Reverse association might be observed after multiple adjustments. COM1: to explain your health concerns to your doctor; COM2: to get enough time in the consultation with your doctor; COM3: to express your personal views and preferences to your doctor; COM4: to ask your doctor questions in the consultation; COM5: to be involved in decisions about your health in dialogue with your doctor; COM6: to recall the information you get from your doctor. GP: general practitioner.

## Discussion

The aim of this study was to report on the level of COM-HL in the adult population in nine European countries and the sociodemographic and medical variables associated with COM-HL. The mean COM-HL level among 18,137 participants from nine countries was 70.2, varying from country to country, but with rather common associated factors across countries. In general, the factors positively associated with COM-HL were age, a better self-perceived social status, being trained in a health profession and an increasing number of visits to the GP. Female sex, financial difficulties, chronic conditions, and an increasing number of visits to specialists were negatively associated with COM-HL.

Although there are no reference values for HLS_19_-COM-P-Q6, our results show that the COM-HL scores ranged from 62.5 to 76.6 out of a possible100 points in the different countries (Table 1). Even if there is room for improvement these country scores and the overall average (70.2) can be considered rather good, since someone who has and answers all items of the HLS_19_-COM-P-Q6 with “easy” has a score of 66.7. The lowest mean score was observed in the German survey, mainly due to item COM2, which almost half of the Germans reported as being (very) difficult (Fig. 1). These difficulties in getting enough time for consultation were shared across countries and call for action across Europe. One reason for these difficulties is probably the significant shortage of physicians^18^ in all countries. However, the item may also be sensitive to the number and type of health issues that patients would like to discuss and the patients’ age, and probably measures other aspects beside COM-HL^16^. The item COM2 ranks second only in the CZ (24%) and FR (24%). In fact, it seems to be slightly more difficult to be involved in decisions concerning one’s health in CZ (25%) or to express one’s point of view and preferences to a physician in FR (26%). These difficulties in involving patients in their own care are not limited to CZ or FR, as these are the two most difficult items at European level after COM2. This finding is consistent with the growing desire for patient empowerment and the call for action to change the physician approaches to improve patient-centered care ^19^.

Several factors were associated with the COM-HL score in the European population. Some of these associations shows a social gradient.

While **age** is negatively associated with general HL in European surveys ^20^ or in other populations^21–23^, the level of COM-HL tended to increase with age, particularly among people between 50 and 64 years of age. Age probably improves the quality of communication because of longer relationships with physicians, or because expectations are lower, or because physicians also spend more time with older people. However, in two countries (AT and SI) the effect of age was reversed. The reasons for this phenomenon are not clear, but it is probably due to factors specific to these countries. In Austria, for example, the younger population has higher values, but this population tends to be healthy and requires little from the health care system. Such a result is not uncommon; in Poland, for example, using another measure showed that communicative health literacy declined with age^24^.

**Women** had lower COM-HL scores. However, this average tendency in Europe was rather small and reached statistically significance only in HU. Such difference was observed for all items of the instrument except COM6 on recalling information received, thus the impact on the overall score is quite conceivable. Many studies have shown that, in general, some physicians communicate less well with women and provide them with less adequate advice and care than with men^25–27^. Studies in Poland^24^ and the Netherlands^28^ found no difference between men and women when measuring COM-HL using a different instrument.

**Self-reported financial difficulties and self-perceived social status** were the most consistent factors associated with COM-HL, as this association was observed in all countries and for all items. These findings recall the importance of the debate of social inequalities, which should be a priority for public decision-makers in health promotion and health care. Indeed, these variables are almost always associated with general HL, regardless of the country or population type^20,29^, underlining the existence of a social gradient, as with general HL^6^. The positive association between a **higher level of education** and COM-HL score was no longer statistically significant after multiple adjustments. This may be explained by the fact that it was associated with self-perceived social status but also significantly associated with all the other variables included in the multivariate model. Moreover, at the item level, an inverse association was observed for two items (COM2 and COM5) after multiple adjustments. One explanation could be that higher education increases expectations in terms of communication, especially to have more time to talk with physicians (COM2) and to be more involved in decision-making about their health (COM5). More research is needed to understand the mediators of the association between education level and COM-HL.

In addition to the variables revealing a social gradient for COM-HL, other variables were associated with it. **Rural residence** was only positively associated with the COM2 (“to get enough time…”). Perhaps rural physicians spend more time with patients they know more during consultations (respectively 7.5 vs. 6.7 min in Iran^30^). Another hypothesis could be that the lower frequency of consultations by people living in rural areas makes the duration of the consultation longer. Different results were observed in France^31^.

**Being trained in a health profession** was positively associated with COM-HL as might be expected, health care professionals also show difficulties in managing information in terms of general HL^32^. The lack of statistically significant association with COM-HL in some countries may be due to lower variability (high levels of COM-HL) in these countries (AT and BE). Training in a health profession was also associated with three of the six items. It seems logical that there should be no association between "time spent in consultation" and being trained in a health profession. On the other hand, we would expect that "expressing preferences…(COM3)" or “ask questions…(COM4) would be easier for people trained in a health profession. The fact that most of these healthcare professionals are subordinate to physicians (nurses, midwives, etc.) could explain these results.

**Having a chronic condition, number of GP and specialist visits** were all associated with COM-HL. These variables may be both determinants and consequences of COM-HL, but it seems logical to recommend improving COM-HL in adults with chronic conditions, as it was done in another cross-sectional study^33^. Similarly, it can be assumed that visiting a GP (compared to a specialist) improves COM-HL. A study in six European countries suggested that the gatekeeping role of GPs is an important factor in physician–patient communication and that in countries where the healthcare system is organized so that GPs do not play this gatekeeping role (BE and DE), their patients expect more comprehensive care than other patients^34^. This could explain why in our study the number of GP visits was not associated with COM-HL in BE and DE. The number of specialist visits was negatively associated with the first three items of HLS_19_-COM-P-Q6. This may reflect the fact that people see several different specialists with less time to express themselves.

Given the potential consequences of COM-HL, it is important to raise awareness and train health professionals to communicate better and more adequately with patients who experience difficulties. A recent multicentre study reported that health professionals are generally unfamiliar with the concept of HL and communication techniques ^35^. This study reinforces the need to educate physicians but also public decision-makers to improve social equity. While COM-HL refers here to the relationship between a patient and his or her physician, it is also highly dependent on the context in which the individual evolves and the way in which the system functions to communicate about health. Communication within the health system as a whole must be considered.

This study has several limitations. Differential item functioning (DIF) exist^16^, with different sample sizes at the country level. The overall mean score should therefore be interpreted with caution. In the same way, the ICC estimate as the between-country variance is inflated by DIF across countries. The lack of harmonization of data collection methods is also a limitation, which we have partly taken into account by adjustment. The data are cross-sectional, and it is sometimes difficult to establish the chronology of onset between the COM-HL score and the variables associated with it. Because non-probability sampling was used in some countries, the risk of type I error is also increased when studying those associations. Despite these limitations, the study has a number of strengths: to its considerable statistical power, this is the first study of COM-HL on a large international scale that highlights levers for action in this area. The HLS_19_-COM-P-Q6 scale^16^ was well accepted with few missing values (3.1% of incomplete scales) among older, less educated, foreign-born people who usually prone to non-response in CATI surveys.

## Conclusion and outlook

The measurement of COM-HL in the European adult population indicates areas for improvement, taking into account the difficulties reported for each item. The determinants of COM-HL are numerous and heterogeneous across countries. However, level of COM-HL varies according to socio-economic status and health care systems within all participating countries, and this study adds to our knowledge of social and health inequalities.

Interventions to improve the communication in healthcare should be a high priority. In particular, supporting health professionals, especially physicians, to spend more time on person-centred communication when needed is important to limit communication inequalities.

## Supplementary Information

Supplementary Information.

## Data Availability

The datasets used and/or analysed during the current study available from the M-POHL consortium (m-pohl@goeg.at) on reasonable request.

## Abbreviations

AT: Austria
BE: Belgium
BG: Bulgaria
COM-HL: Communicative Health Literacy
CZ: Czech Republic
DE: Germany
DIF: Differential item functioning
DK: Denmark
FR: France
GP: General practitioner
HL: Health literacy
HLS_19_: Health Literacy Survey 2019–2021
HU: Hungary
ICC: Intra-Class Coefficient
M-POHL: WHO Action Network on Measuring Population and Organizational Health Literacy
SD: Standard deviation
SI: Slovenia
