# Supplementary material for: Communicative health literacy and associated variables in nine European countries: results from the HLS19 survey
Source: Sci Rep. 2024 Dec 4;14:30245. doi: 10.1038/s41598-024-79327-w (PMC11618785; doi:10.1038/s41598-024-79327-w)
Supplement: Supplementary file 1 — Supplementary Information. [file 41598_2024_79327_MOESM1_ESM.pdf]

SUPPLEMENTARY DATA

Table S1: Socio-demographic and medical characteristics of the included population vs the excluded population (with at least one missing HLS_{19}-COM-P-Q6 item).

**Characteristic**	**Excluded pop**, N = 585[1]	**Included pop**, N = 18,137[1]	**Overall**, N = 18,722[1]	**p-value**[2]
Age (years)	55 (18)	49 (18)	49 (18)	<0.001
Unknown	*2*	*22*	*24*	
Sex (female)	287 (49%)	9,331 (51%)	9,618 (51%)	0.4
Unknown	*0*	*6*	*6*	
Rural living	226 (39%)	6,257 (35%)	6,484 (35%)	0.13
Unknown	*0*	*1*	*1*	
Birth abroad	43 (7.4%)	1,182 (6.5%)	1,225 (6.6%)	0.5
Unknown	*1*	*13*	*14*	
Level of education				<0.001
≤ college	193 (33%)	3,590 (20%)	3,783 (20%)	
Upper secondary education	162 (28%)	5,258 (29%)	5,419 (29%)	
≤ Bachelor's degree	55 (9.5%)	3,829 (21%)	3,885 (21%)	
≥ Bachelor's degree	174 (30%)	5,397 (30%)	5,570 (30%)	
Unknown	*2*	*64*	*66*	
Trained in a health profession	113 (19%)	2,604 (14%)	2,717 (15%)	0.029
Unknown	*1*	*37*	*39*	
Financial difficulties (ability to pay bills)				<0.001

**Characteristic**	**Excluded pop**, N = 585[1]	**Included pop**, N = 18,137[1]	**Overall**, N = 18,722[1]	**p-value**[2]
Very difficult	48 (8.0%)	616 (3.4%)	662 (3.6%)	
Difficult	152 (27%)	3,815 (21%)	3,967 (21%)	
Easy	275 (48%)	9,290 (52%)	9,565 (52%)	
Very easy	95 (17%)	4,227 (24%)	4,322 (23%)	
Unknown	*17*	*189*	*206*	
Self-perceived social status	5.47 (1.88)	5.88 (1.65)	5.87 (1.66)	0.003
Unknown	*57*	*382*	*439*	
Chronic condition	264 (46%)	8,328 (46%)	8,592 (46%)	>0.9
Unknown	*15*	*75*	*91*	
Number of visits to the GP				<0.001
0	201 (36%)	3,801 (21%)	4,002 (22%)	
1	126 (22%)	3,855 (21%)	3,981 (22%)	
2	94 (17%)	3,414 (19%)	3,508 (19%)	
≥3	143 (25%)	6,882 (38%)	7,025 (38%)	
Unknown	*21*	*185*	*206*	
Number of visits to a specialist				0.035
0	287 (51%)	7,589 (42%)	7,877 (42%)	
1	107 (19%)	3,861 (21%)	3,969 (21%)	
2	67 (12%)	2,667 (15%)	2,734 (15%)	
≥3	103 (18%)	3,884 (22%)	3,987 (21%)	
Unknown	*20*	*135*	*156*	
Mode of data collection				<0.001
CAPI/PAPI	226 (39%)	4,279 (24%)	4,504 (24%)	

Characteristic	**Excluded pop**, N = 585[1]	**Included pop**, N = 18,137[1]	**Overall**, N = 18,722[1]	**p-value**[2]
CATI	246 (42%)	4,422 (24%)	4,668 (25%)	
CAWI	114 (19%)	9,436 (52%)	9,550 (51%)	

[1] Mean (SD); n (%); [2] Wilcoxon rank-sum test for complex survey samples; chi-squared test with Rao & Scott's second-order correction; pop: population; CAPI = computer-assisted personal interviews; CATI = computer-assisted telephone interviews; CAWI = computer-assisted web interviews; GP: general practitioner; PAPI = paper-assisted personal interviews; GP: general practitioner.

Table S2: Factors associated with exclusion (i.e. at least one missing HLS_{19}-COM-P-Q6 item)

Variables	cOR	aOR*	p-value
**Age (years)**			
≤34	1	1	
]34 ; 50]	1.15 (0.84, 1.58)	1.36 (0.94, 1.96)	0.10
]50 ; 64]	1.61 (1.17, 2.21)	1.90 (1.32, 2.73)	**<0.001**
> 64	2.04 (1.43, 2.90)	2.72 (1.79, 4.14)	**<0.001**
**Sex**			
Male	1	1	
Female	0.89 (0.70, 1.12)	0.81 (0.62, 1.06)	0.12
**Area of residence**			
Urban	1	1	
Rural	1.07 (0.84, 1.38)	1.04 (0.80, 1.36)	0.8
**Birth abroad**			
No	1	1	
Yes	1.41 (0.95, 2.08)	1.61 (1.03, 2.50)	**0.035**
**Level of education**			
≤ college	1	1	
Upper secondary education	0.53 (0.39, 0.72)	0.64 (0.45, 0.89)	**0.008**
≤ Bachelor's degree	0.49 (0.33, 0.72)	0.51 (0.32, 0.83)	**0.007**
≥ Bachelor's degree	0.60 (0.42, 0.85)	0.71 (0.46, 1.07)	0.10
**Financial difficulties**			
Very easy	1	1	
Easy	1.10 (0.76, 1.58)	0.99 (0.67, 1.45)	>0.9
Difficult	1.30 (0.84, 2.00)	1.14 (0.72, 1.81)	0.6
Very difficult	2.44 (1.40, 4.27)	1.85 (1.04, 3.31)	**0.037**
**Self-perceived social status**	0.88 (0.81, 0.96)	0.91 (0.84, 0.99)	**0.034**
**Trained in a health profession**	0.94 (0.70, 1.28)	0.81 (0.58, 1.12)	0.2
**Chronic condition**			
No	1	1	
Yes	1.00 (0.80, 1.26)	0.98 (0.75, 1.28)	>0.9
**Number of visits to the GP**			
0	1	1	
1	0.77 (0.57, 1.04)	0.66 (0.47, 0.93)	**0.016**
2	0.65 (0.44, 0.97)	0.56 (0.37, 0.82)	**0.003**
≥ 3	0.48 (0.36, 0.64)	0.36 (0.25, 0.52)	**<0.001**
**Number of visits to a specialist**			

Variables	cOR	aOR*	p-value
0	1	1	
1	0.73 (0.54, 0.97)	0.89 (0.64, 1.23)	0.5
2	0.68 (0.43, 1.08)	0.92 (0.57, 1.49)	0.7
≥ 3	0.71 (0.53, 0.95)	0.94 (0.65, 1.35)	0.7
**Mode of data collection**			
CAPI/PAPI	1	1	
CATI	30.6 (3.94, 237)	24.6 (3.16, 191)	**0.002**
CAWI	0.54 (0.34, 0.85)	0.64 (0.39, 1.06)	0.086

* All models are adjusted for country; aOR: adjusted odds ratio; cOR: crude odds ratio; CAPI = computer-assisted personal interviews; CATI = computer-assisted telephone interviews; CAWI = computer-assisted web interviews; CI 95%: 95% confidence interval; GP: general practitioner; PAPI = paper-assisted personal interviews.

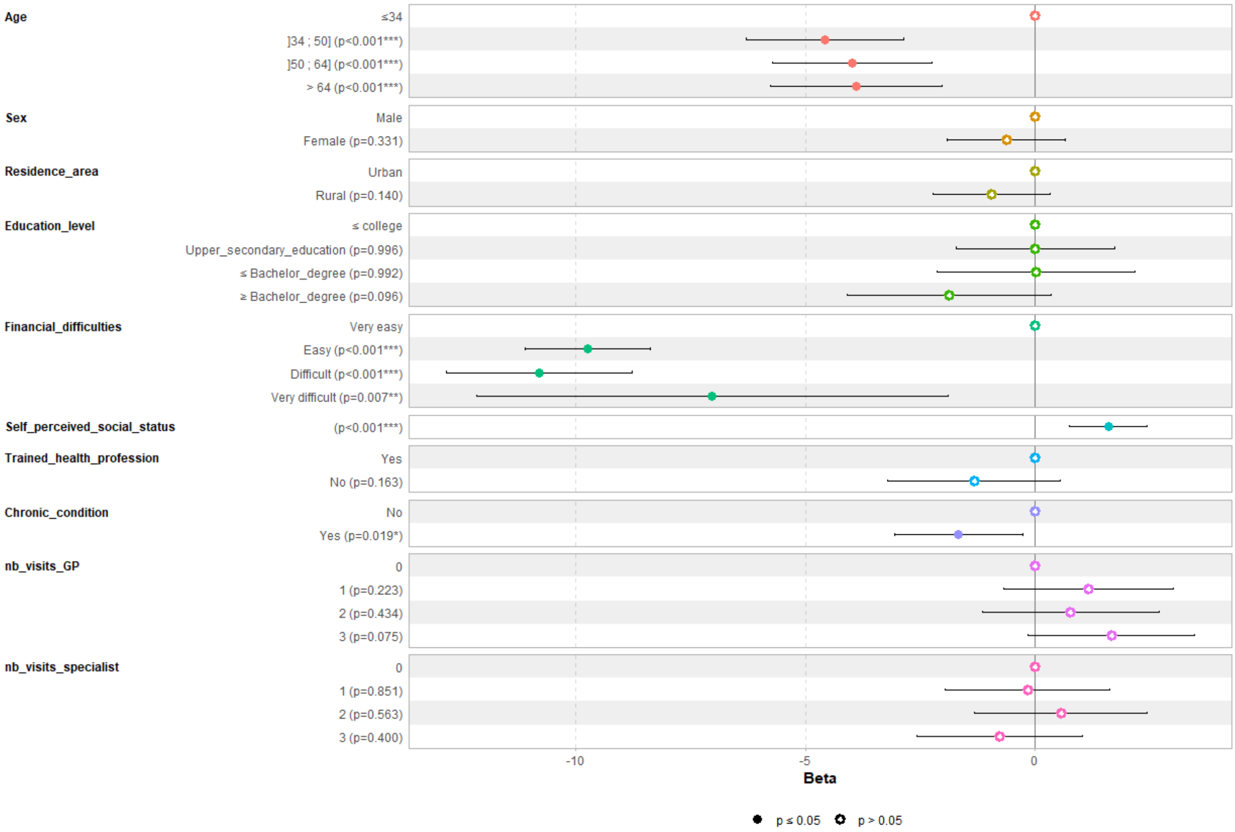

Figure S1: Factors associated with the COM-HL score in Austria.

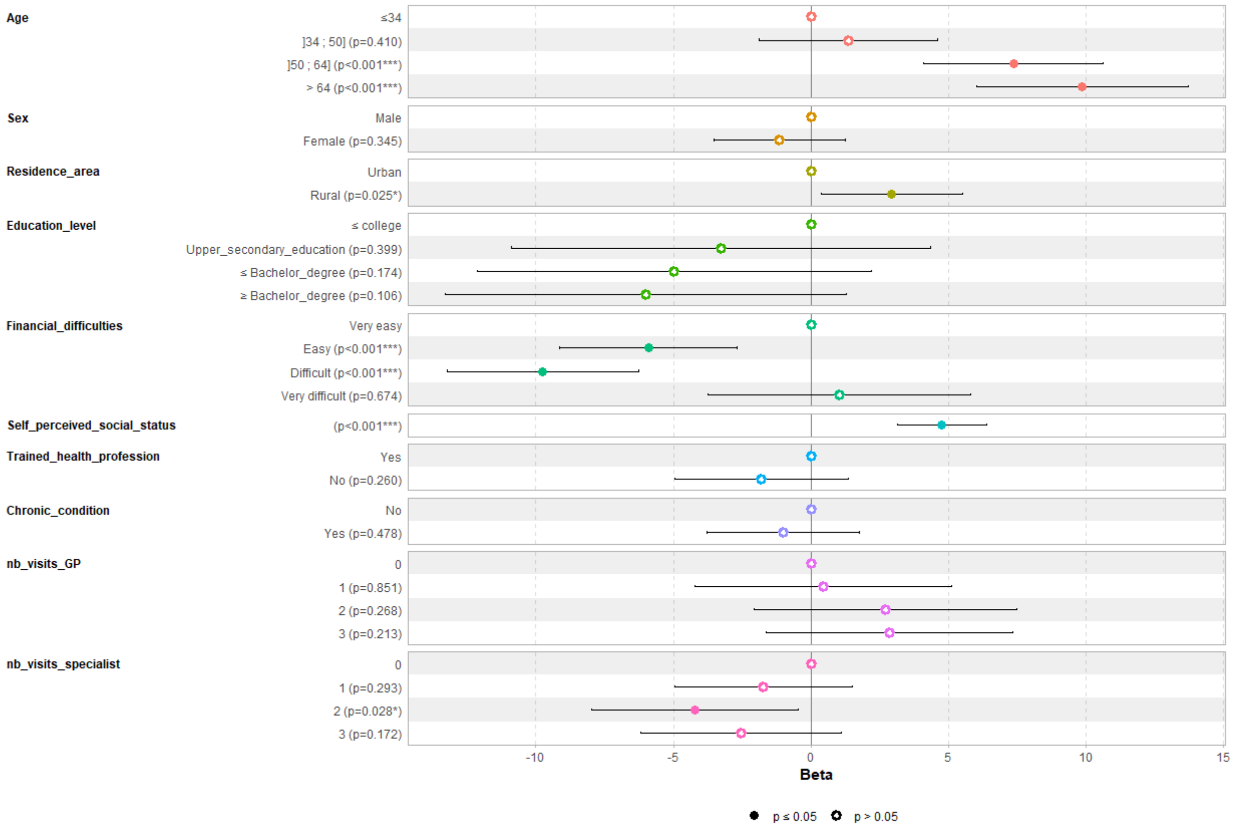

Figure S2: Factors associated with the COM-HL score in Belgium.

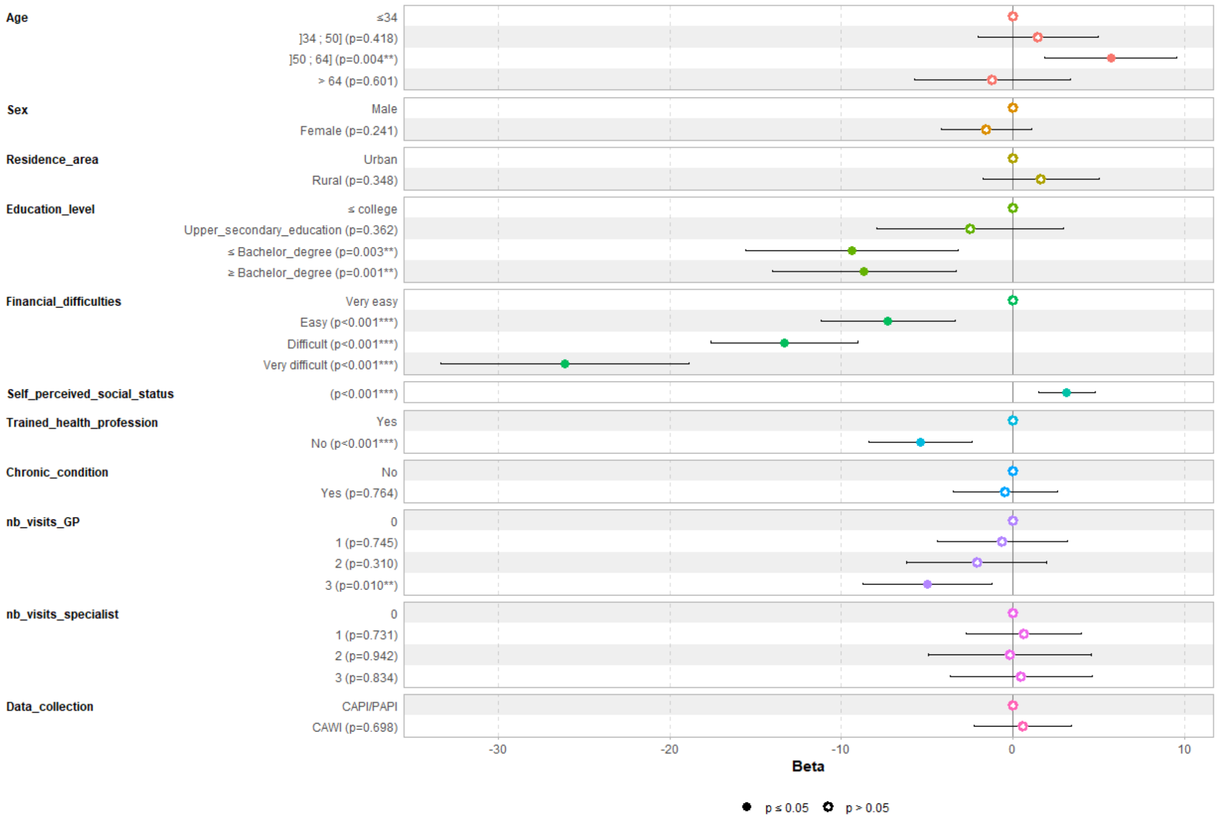

Figure S3: Factors associated with the COM-HL score in Bulgaria.

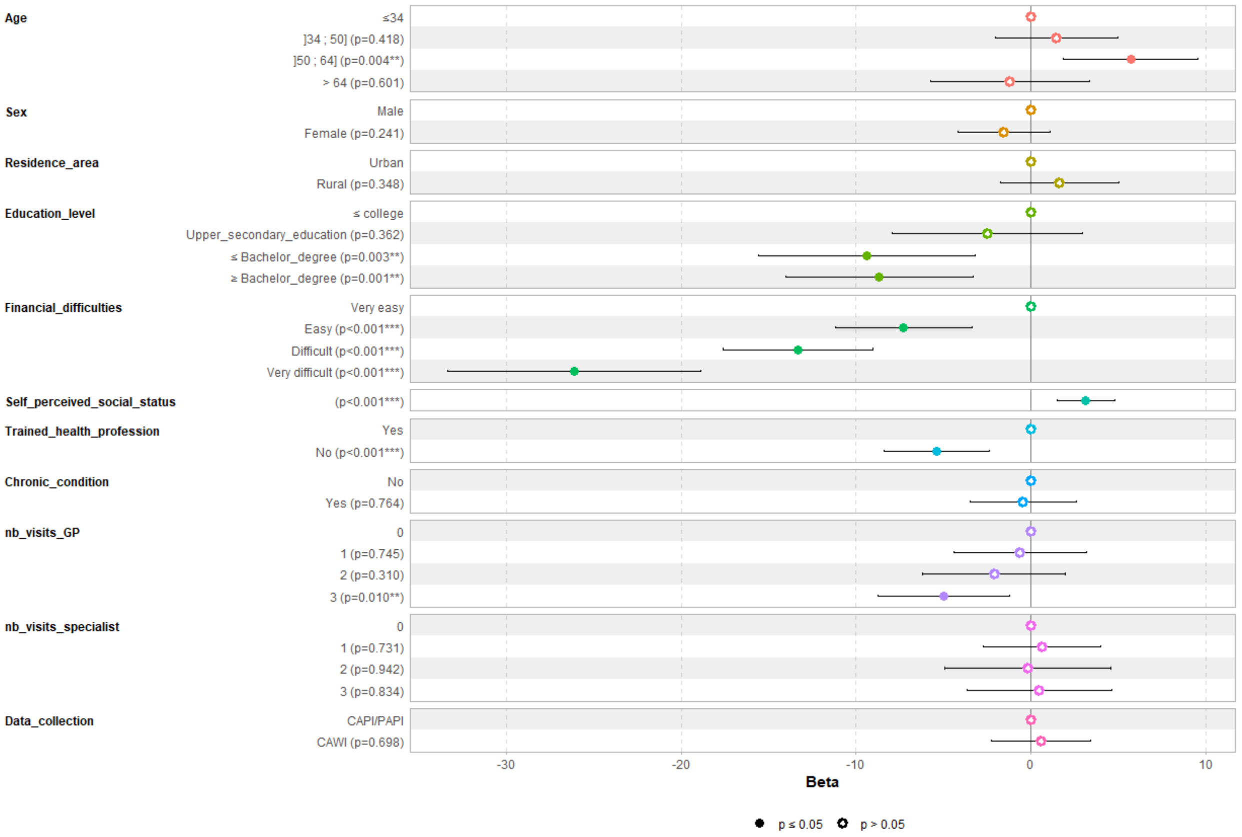

Figure S4: Factors associated with the COM-HL score in the Czech Republic.

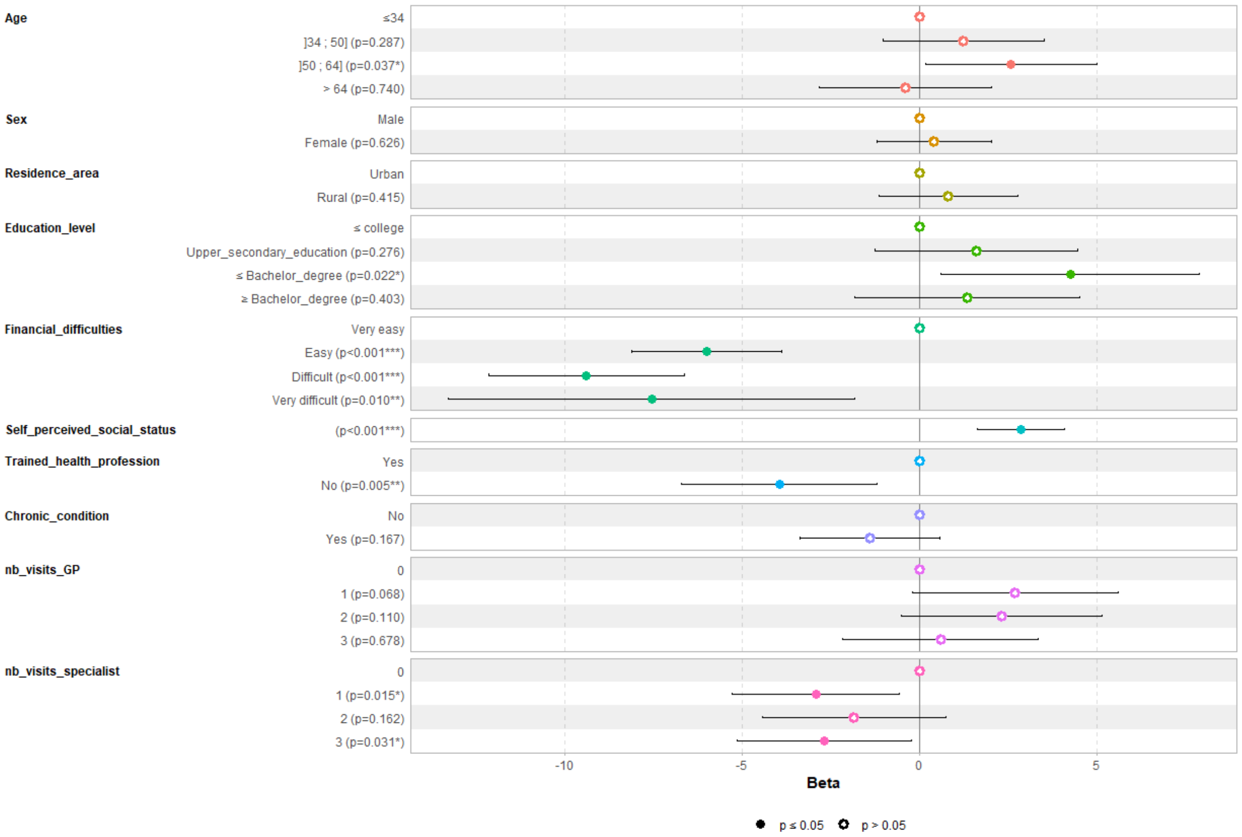

Figure S5: Factors associated with the COM-HL score in Germany.

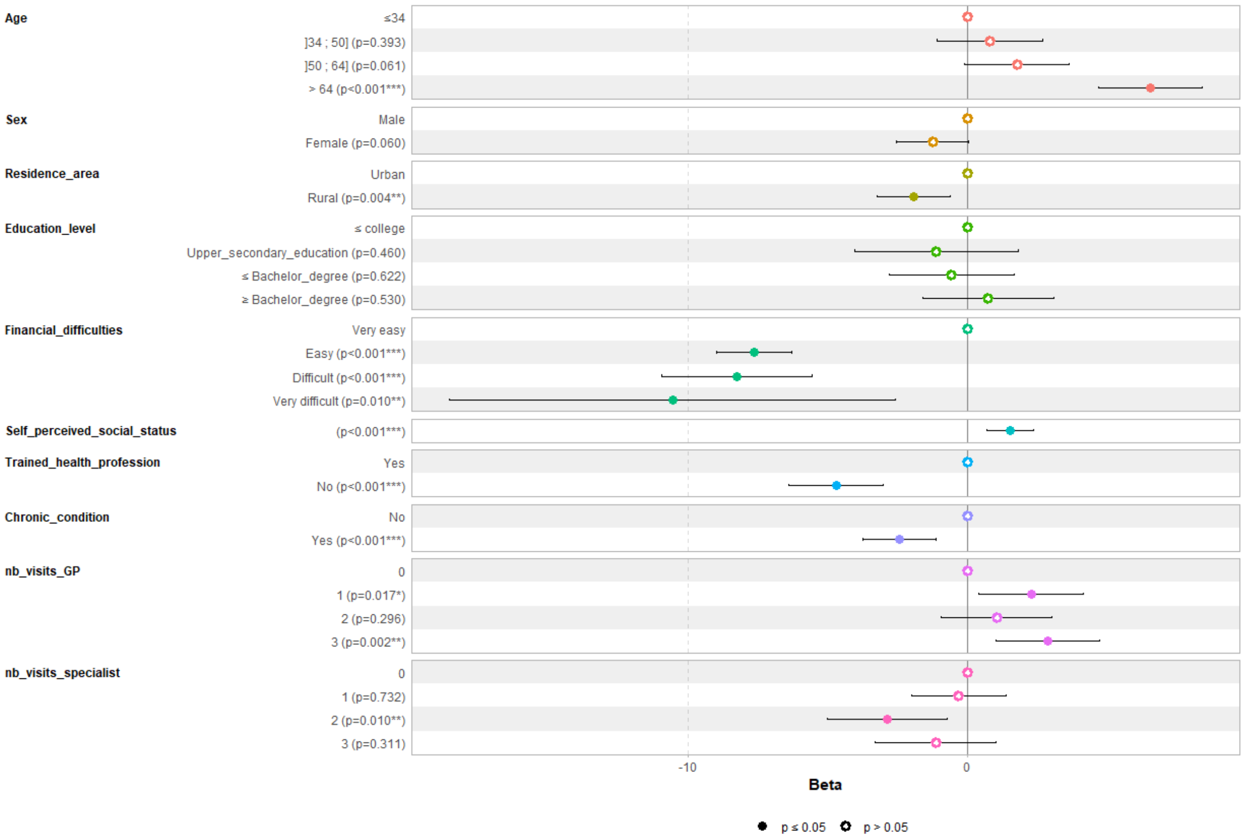

Figure S6: Factors associated with the COM-HL score in Denmark.

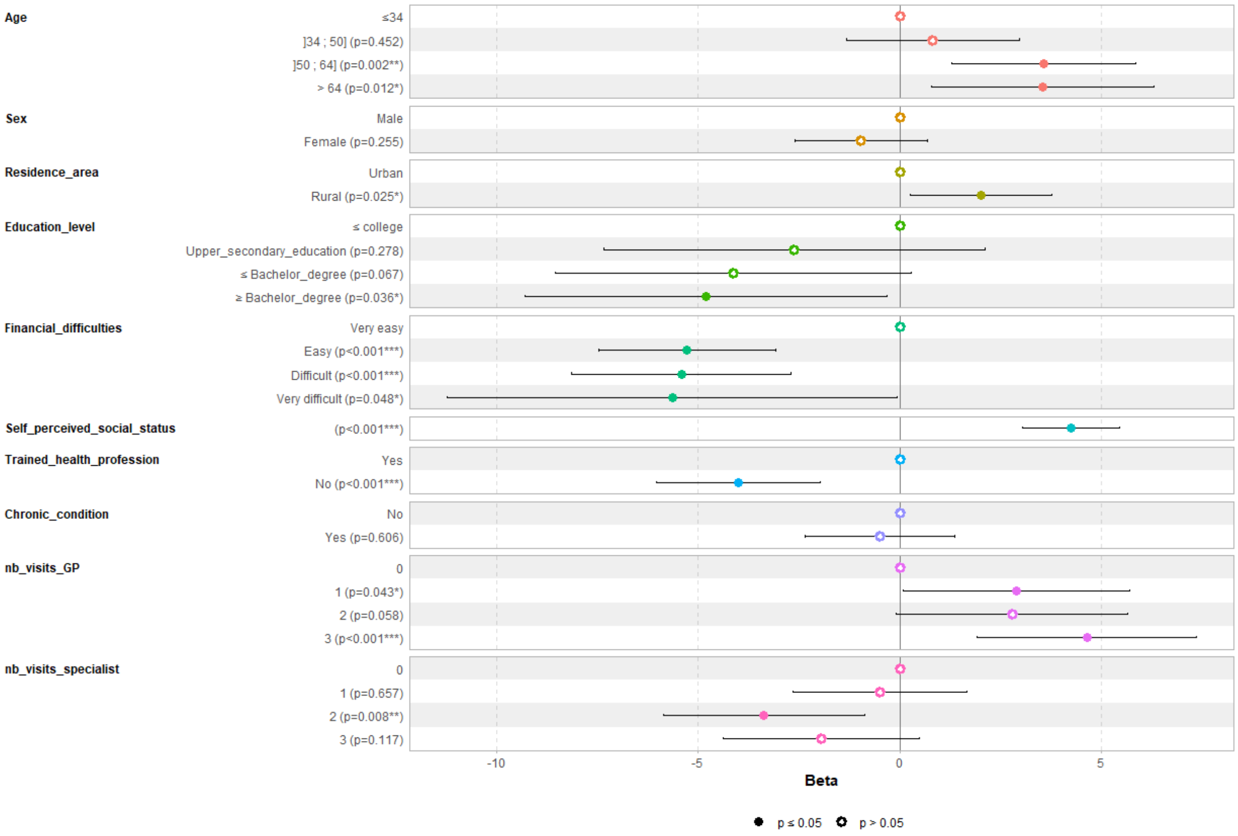

Figure S7: Factors associated with the COM-HL score in France.

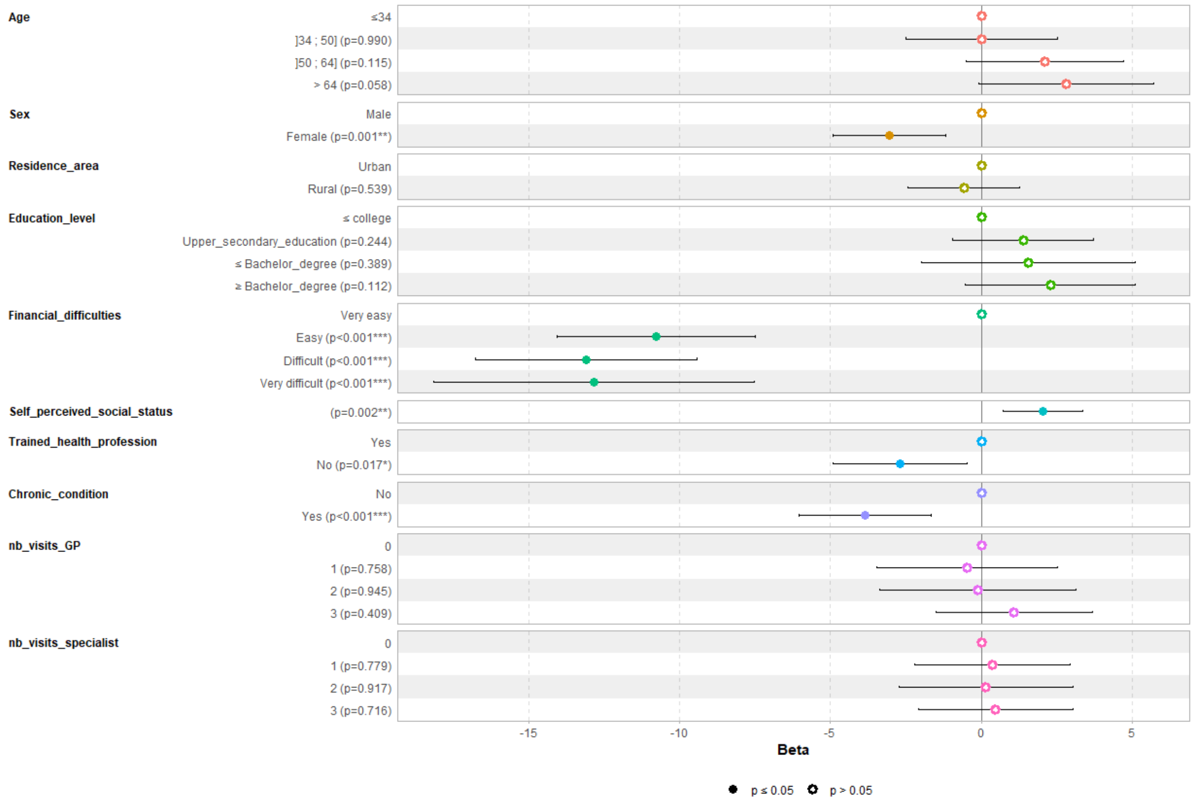

Figure S8: Factors associated with the COM-HL score in Hungary.

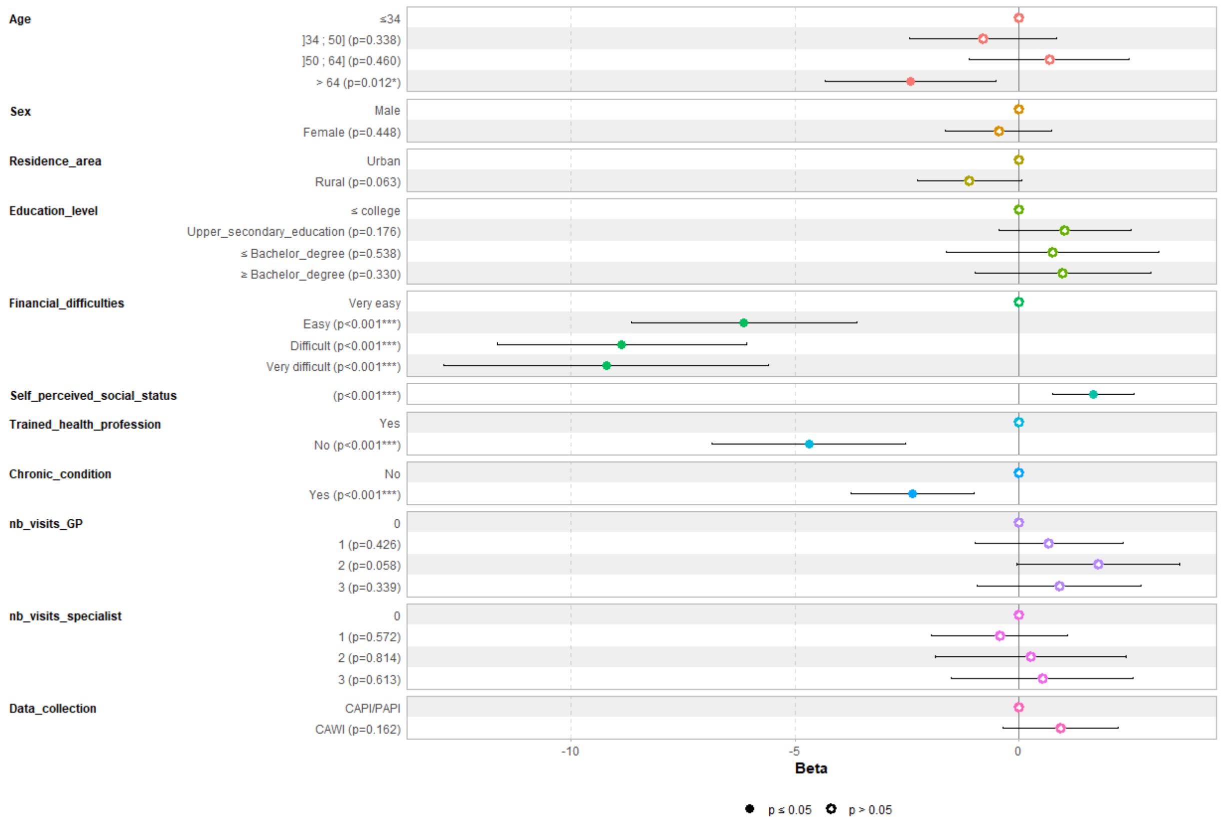

Figure S9: Factors associated with the COM-HL score in Slovenia.

Table S3: Variables associated with "very difficult" or "difficult" responses for the "...to explain your health concern to your doctor?" item (COM1)

**Variables**	Initial model*		Final multivariate model*	
	Crude OR	*p-value*	Adjusted OR	*p-value*
**Age (years)****				
≤34	Ref		Ref	
]34 ; 50]	0.87	0.04	0.90	0.338
]50 ; 64]	0.78	<0.001	0.71	**<0.001**
> 64	0.69	<0.001	0.62	**0.004**
**Sex****				
Male	Ref		Ref	
Female	1.28	<0.001	1.27	**0.002**
**Area of residence**				
Urban	Ref		Ref	
Rural	1.09	0.13	1.08	0.203
**Level of education****				
≤ Lower secondary eductaion	Ref		Ref	
Upper secondary education	0.89	0.15	1.14	0.369
≤ Bachelor's degree	0.77	<0.001	1.05	0.835
≥ Bachelor's degree	0.56	<0.001	0.91	0.454
**Financial difficulties (ability to pay bill)**				
Very easy	Ref		Ref	
Easy	1.79	<0.001	1.50	**<0.001**
Difficult	3.27	<0.001	2.31	**<0.001**
Very difficult	4.41	<0.001	2.71	**<0.001**
**Self-perceived social status**	0.62	<0.001	0.87	**<0.001**
**Trained in a health profession**				
Yes	Ref		Ref	
No	1.33	<0.001	1.26	**0.006**
**Chronic condition**				
No	Ref		Ref	
Yes	0.42	<0.001	1.40	**<0.001**
**Number of visits to the GP**				
0	Ref		Ref	
1	0.87	0.09	0.86	0.087
2	1.02	0.85	0.94	0.505
≥ 3	1.16	0.04	0.86	0.076
**Number of visits to a specialist**				
0	Ref		Ref	
1	1.08	0.27	1.11	0.184
2	1.19	0.03	1.13	0.158
≥ 3	1.51	<0.001	1.31	**<0.001**
**Country effect (ICC)**	3%***		7%	

*All models account for the clustering (country) effect and are adjusted for mode of data collection; **Random slope at country level was added in the multilevel model; GP: general practitioner; ***The initial model with only intercept and a random effect (for country) was significant (p<0.001).

Table S4: Variables associated with "very difficult" or "difficult" responses for the "... to get enough time in the consultation with your doctor? " item (COM2)

**Variables**	Initial model*		Final multivariate model*	
	Crude OR	*p-value*	Crude OR	*p-value*
**Age (years)****				
≤34	Ref		Ref	
]34 ; 50]	1.14	<0.001	1.13	0.292
]50 ; 64]	1.02	0.64	0.94	0.554
> 64	0.84	<0.001	0.75	**0.032**
**Sex**				
Male	Ref		Ref	
Female	1.26	<0.001	1.20	**<0.001**
**Area of residence****				
Urban	Ref		Ref	
Rural	0.9	0.01	0.79	**0.035**
**Level of education**				
≤ Lower secondary eductaion	Ref		Ref	
Upper secondary education	1.02	0.67	1.18	**0.008**
≤ Bachelor's degree	1.15	0.03	1.28	**<0.001**
≥ Bachelor's degree	1.14	0.03	1.50	**<0.001**
**Financial difficulties (ability to pay bill)**				
Very easy	Ref		Ref	
Easy	1.54	<0.001	1.46	**<0.001**
Difficult	2.3	<0.001	2.05	**<0.001**
Very difficult	2.5	<0.001	2.12	**<0.001**
**Self-perceived social status****	0.80	<0.001	0.90	**<0.001**
**Trained in a health profession**				
Yes	Ref		Ref	
No	0.92	0.12	1.00	0.968
**Chronic condition****				
No	Ref		Ref	
Yes	1.27	<0.001	1.22	**0.014**
**Number of visits to the GP**				
0	Ref		Ref	
1	0.91	0.10	0.84	**0.004**
2	1.03	0.63	0.89	0.060
≥ 3	1.04	0.45	0.83	**0.002**
**Number of visits to a specialist**				
0	Ref		Ref	
1	1.2	<0.001	1.18	**<0.001**
2	1.24	<0.001	1.25	**<0.001**
≥ 3	1.39	<0.001	1.29	**<0.001**
**Country effect (ICC)**	6%***		21%	

*All models account for the clustering (country) effect and are adjusted for mode of data collection; **Random slope at country level was added in the multilevel model; GP: general practitioner; ***The initial model with only intercept and a random effect (for country) was significant (p<0.001).

Table S5: Variables associated with "very difficult" or "difficult" responses for the "... to express your personal views and preferences to your doctor? " item (COM3)

**Variables**	Initial model*		Final multivariate model*	
	Crude OR	*p-value*	Crude OR	*p-value*
**Age (years)****				
≤34	Ref		Ref	
]34 ; 50]	0.85	<0.001	0.87	0.195
]50 ; 64]	0.71	<0.001	0.66	**0.001**
> 64	0.67	<0.001	0.62	**0.001**
**Sex****				
Male	Ref		Ref	
Female	1.31	<0.001	1.28	**<0.001**
**Area of residence**				
Urban	Ref		Ref	
Rural	0.97	0.56	0.99	0.763
**Level of education**				
≤ Lower secondary eductaion	Ref		Ref	
Upper secondary education	0.9	0.09	1.02	0.719
≤ Bachelor's degree	0.84	0.02	1.05	0.512
≥ Bachelor's degree	0.77	<0.001	1.07	0.345
**Financial difficulties (ability to pay bill)**				
Very easy	Ref		Ref	
Easy	1.62	<0.001	1.41	**<0.001**
Difficult	2.51	<0.001	1.85	**<0.001**
Very difficult	3.44	<0.001	2.20	**<0.001**
**Self-perceived social status**	0.67	<0.001	0.87	**<0.001**
**Trained in a health profession**				
Yes	Ref		Ref	
No	1.1	0.10	1.11	0.100
**Chronic condition****				
No	Ref		Ref	
Yes	1.25	<0.001	1.23	**0.002**
**Number of visits to the GP**				
0	Ref		Ref	
1	0.93	0.28	0.92	0.238
2	0.99	0.99	0.97	0.644
≥ 3	1.07	0.17	0.90	0.106
**Number of visits to a specialist**				
0	Ref		Ref	
1	0.96	0.51	0.96	0.513
2	1.17	0.01	1.19	**0.009**
≥ 3	1.28	<0.001	1.19	**0.006**
**Country effect (ICC)**	5%***		9%	

*All models account for the clustering (country) effect and are adjusted for mode of data collection; **Random slope at country level was added in the multilevel model; GP: general practitioner; ***The initial model with only intercept and a random effect (for country) was significant (p<0.001).

.

Table S6: Variables associated with "very difficult" or "difficult" responses for the "... to ask your doctor questions in the consultation? " item (COM4)

**Variables**	Initial model*		Final multivariate model*	
	Crude OR	*p-value*	Crude OR	*p-value*
**Age (years)****				
≤34	**Ref**		Ref	
]34 ; 50]	0.79	<0.001	0.78	**<0.001**
]50 ; 64]	0.68	<0.001	0.60	**<0.001**
> 64	0.74	<0.001	0.68	**0.023**
**Sex**				
Male	Ref		Ref	
Female	1.19	<0.001	1.13	**0.021**
**Area of residence**				
Urban	Ref		Ref	
Rural	0.97	0.54	0.97	0.555
**Level of education**				
≤ Lower secondary eductaion	Ref		Ref	
Upper secondary education	0.88	0.11	1.05	0.524
≤ Bachelor's degree	0.82	0.02	1.10	0.302
≥ Bachelor's degree	0.75	<0.001	1.18	0.072
**Financial difficulties (ability to pay bill)**				
Very easy	Ref		Ref	
Easy	1.61	<0.001	1.49	**<0.001**
Difficult	2.74	<0.001	2.07	**<0.001**
Very difficult	3.98	<0.001	2.59	**<0.001**
**Perception of level in the society****	0.65	<0.001	0.84	**<0.001**
**Previous training in a health profession**				
Yes	Ref		Ref	
No	1.2	0.01	1.15	0.077
**Chronic illness****				
No	Ref		Ref	
Yes	1.32	<0.001	1.36	**<0.001**
**Number of visits to the GP**				
0	Ref		Ref	
1	0.78	<0.001	0.83	**0.019**
2	0.81	0.01	0.81	**0.014**
≥ 3	0.93	0.29	0.79	**0.002**
**Number of visits to a specialist**				
0	Ref		Ref	
1	0.84	0.01	0.90	0.160
2	1.02	0.78	1.07	0.403
≥ 3	1.17	0.01	1.12	0.153
**Country effect (ICC)**	5%***		10%	

*All models account for the clustering (country) effect and are adjusted for mode of data collection; **Random slope at country level was added in the multilevel model; GP: general practitioner; ***The initial model with only intercept and a random effect (for country) was significant (p<0.001).

Table S7: Variables associated with "very difficult" or "difficult" responses for the "... to be involved in decisions about your health in dialogue with your doctor? " item (COM5)

**Variables**	Initial model*		Final multivariate model*	
	Crude OR	*p-value*	Crude OR	*p-value*
**Age (years)****				
≤34	Ref		Ref	
]34 ; 50]	0.83	<0.001	0.95	0.660
]50 ; 64]	0.68	<0.001	0.70	**<0.001**
> 64	0.72	<0.001	0.71	**0.048**
**Sex**				
Male	Ref		Ref	
Female	1.22	<0.001	1.15	**0.002**
**Area of residence**				
Urban	Ref		Ref	
Rural	0.94	0.24	0.95	0.263
**Level of education****				
≤ Lower secondary eductaion	Ref		Ref	
Upper secondary education	0.87	0.03	1.05	0.669
≤ Bachelor's degree	0.75	<0.001	1.06	0.622
≥ Bachelor's degree	0.78	<0.001	1.31	**0.014**
**Financial difficulties (ability to pay bill)**				
Very easy	Ref		Ref	
Easy	1.62	<0.001	1.42	**<0.001**
Difficult	2.57	<0.001	1.87	**<0.001**
Very difficult	3.61	<0.001	2.21	**<0.001**
**Self-perceived social status**	0.64	<0.001	0.84	**<0.001**
**Trained in a health profession**				
Yes	Ref		Ref	
No	1.26	<0.001	1.25	**<0.001**
**Chronic condition****				
No	Ref		Ref	
Yes	1.26	<0.001	1.27	**0.002**
**Number of visits to the GP****				
0	Ref		Ref	
1	0.9	0.10	0.87	0.103
2	0.89	0.09	0.83	**0.046**
≥ 3	0.27	0.96	0.87	0.194
**Number of visits to a specialist**				
0	Ref		Ref	
1	0.95	0.34	0.98	0.704
2	1.04	0.54	1.06	0.428
≥ 3	1.15	0.01	1.05	0.428
**Country effect (ICC)**	5%***		11%	

*All models account for the clustering (country) effect and are adjusted for mode of data collection; **Random slope at country level was added in the multilevel model; GP: general practitioner; ***The initial model with only intercept and a random effect (for country) was significant (p<0.001).

Table S8: Variables associated with "very difficult" or "difficult" responses for the "... to recall the information you get from your doctor? " item (COM6)

**Variables**	Initial model*		Final multivariate model*	
	Crude OR	*p-value*	Crude OR	*p-value*
**Age (years)****				
≤34	Ref		Ref	
]34 ; 50]	0.92	0.15	0.93	0.653
]50 ; 64]	0.75	<0.001	0.63	**0.008**
> 64	0.95	0.39	0.78	0.096
**Sex**				
Male	Ref		Ref	
Female	1.12	0.01	1.05	0.264
**Area of residence**				
Urban	Ref		Ref	
Rural	1.06	0.17	1.05	0.320
**Level of education****				
≤ Lower secondary eductaion	Ref		Ref	
Upper secondary education	0.7	<0.001	0.88	0.300
≤ Bachelor's degree	0.67	<0.001	0.95	0.832
≥ Bachelor's degree	0.54	<0.001	0.94	0.738
**Financial difficulties (ability to pay bill)**				
Very easy	Ref		Ref	
Easy	1.84	<0.001	1.57	**<0.001**
Difficult	2.79	<0.001	1.99	**<0.001**
Very difficult	3.93	<0.001	2.42	**<0.001**
**Self-perceived social status**	0.65	<0.001	0.87	**<0.001**
**Trained in a health profession**				
Yes	Ref		Ref	
No	1.59	<0.001	1.56	**<0.001**
**Chronic condition**				
No	Ref		Ref	
Yes	1.38	<0.001	1.29	**0.002**
**Number of visits to the GP****				
0	Ref		Ref	
1	1.05	0.52	1.13	0.259
2	1.16	0.04	1.13	0.214
≥ 3	1.41	<0.001	1.22	0.055
**Number of visits to a specialist**				
0	Ref		Ref	
1	1.01	0.89	0.96	0.582
2	1.18	0.01	1.08	0.323
≥ 3	1.38	<0.001	1.17	**0.023**
**Country effect (ICC)**	8%***		11%	

*All models account for the clustering (country) effect and are adjusted for mode of data collection; **Random slope at country level was added in the multilevel model; GP: general practitioner; ***The initial model with only intercept and a random effect (for country) was significant (p<0.001).
